# Your ID, please? The effect of facemasks and makeup on perceptions of age of young adult female faces

**DOI:** 10.1002/acp.3923

**Published:** 2022-02-06

**Authors:** Hannah Davis, Janice Attard‐Johnson

**Affiliations:** ^1^ Department of Psychology, Faculty of Science and Technology Bournemouth University Poole UK

**Keywords:** age estimation, cosmetics, COVID‐19, face perception, facemasks

## Abstract

During the COVID‐19 pandemic, wearing facemasks was mandatory in the United Kingdom except for individuals with medical exemptions. Facemasks cover the full lower half of the face; however, the effect of facemasks on age perception is not yet known. The present study examined whether age estimation accuracy of unfamiliar young adult women is impaired when the target is wearing a facemask. This study also examined whether makeup, which has previously been shown to increase error bias, further impairs age estimation accuracy when paired with a facemask. The findings indicate that both facemasks and makeup tend to result in overestimation of the young women's age compared to neutral faces, but the combination of both is not additive. Individual level analysis also revealed large individual differences in age estimation accuracy ranging from estimates within 1 year of the target's actual age, and age estimates which deviated by up to 20 years.

## 1 INTRODUCTION

Accurate age estimation of strangers is paramount in situations relating to forensic age identification in criminal investigations (Thorley, 2018) and age verification for age restricted sales (Willner & Rowe, 2001). Laboratory studies demonstrate that age estimates are moderately accurate with errors within the range of 3–5 years in adults (Sörqvist & Eriksson, 2007). However, factors which impair age estimation accuracy by up to 8 years have also been identified (Clifford et al., 2018; Dehon & Brédart, 2001; Voelkle et al., 2012; for reviews see Moyse, 2014; Rhodes, 2009). One example is the use of sunglasses to obscure the eye region and disguise visual cues of age within this region (Thorley, 2021). However, the effect of obscuring other facial features on age perception is less known.

During the COVID‐19 pandemic in the UK, individuals were mandated to wear a facemask when indoors with members of different households or with strangers, when on public transport, in shopping centres and supermarkets, and on hospitality premises (Department of Health and Social Care, 2020). Although use of facemasks potentially presents a challenge for salespeople selling alcohol, knives or other age‐restricted items, the effect of facemasks on age estimation accuracy is not known. The challenge is further compounded by individuals who may unintentionally or deliberately attempt to appear older when wearing a facemask by also applying facial cosmetics to alter the visual cues of the visible facial features. Stores are encouraged to use the ‘*Challenge 25’* Policy and should therefore request identification to confirm that the buyers are over the age of 18 when they perceive an individual to be under the age of 25. However, the extent to which facemasks can impair age estimation accuracy, if at all, is not clear. Although government mandates to wear facemasks will gradually lift as the pandemic subsides, some people will choose to continue to wear facemasks as evident from some cultures during pre‐pandemic times (Lau et al., 2010). Therefore, the question of whether facemasks, and makeup, distort age perception will likely continue to be relevant even after restrictions have been lifted.

The perceived age of women is typically found to be less accurate compared to men (Voelkle et al., 2012), with younger faces frequently overestimated and older faces underestimated (Voelkle et al., 2012; Watson et al., 2016). The discrepancy between men and women may be attributed, at least in part, to the use of cosmetics. Women are more likely than men to apply cosmetics to alter their physical appearance usually with the desired effect of appearing more attractive and decreasing negative self‐perception (Korichi & Pelle‐de‐Queral, 2008). Previous studies revealed that faces wearing cosmetics are judged as healthier (Nash et al., 2006) and more attractive (Mulhern et al., 2003), and the latter correlates negatively with perceived age (Henss, 1991). Although the effect of cosmetics on human age estimation has not been extensively researched, a small set of studies support the notion that cosmetics alter the perception of age (Russell, 2003; Russell et al., 2019).

Cosmetics alter the perception of specific facial features, which act as visual cues for age. One cue is facial contrast, which comprises the colour differences and luminance in the skin and between the facial features. Facial contrast changes with age, and faces with increased facial contrast (e.g. darker eyebrows and lips) are perceived to be younger compared to those with decreased facial contrast (Porcheron et al., 2013). Consequently, makeup, which is applied to darken the lips, increase skin luminance, and increase the colour contrast of the eyes and eyebrows, work to increase facial contrast and result in younger looking faces (Russell et al., 2019). Makeup applied to different facial features also has different effects on age perception. For example, makeup applied to the eyes and eyebrows has a stronger impact on age perception compared to application to the lips (Russell et al., 2019). Additionally, the effects of these manipulations to the appearance of the face are dependent on the target age (Egan & Cordan, 2009; Russell et al., 2019). Specifically, photographs of faces depicting 40 and 50‐year‐old women are perceived on average 1.5 years younger when wearing full makeup, while 20‐year‐old women appear 1.4 years older with identical makeup (Russell et al., 2019). Although these estimation errors are relatively modest, other studies have reported a larger range of errors of up to 20 years older or younger than actual age (Dayan et al., 2015; Fink et al., 2006).

The difference in the strength of the effects when applying makeup to lips compared to the eye region suggests that some facial features may play a more prominent role in providing visual cues to age. Only a small set of studies have examined the effect of obscuring different facial features on age perception. In those studies, obscuring head shape and hair did not decrease accuracy (George & Hole, 1995), but obscuring the eye region led to a greater reduction in accuracy compared to disguising the hair and forehead or no disguise at all (Thorley, 2021). Furthermore, a recent eye‐tracking study suggests that the central triangle (collectively eyes, nose, and mouth) may be important for age estimation (Liao et al., 2020). It is therefore not inconceivable that obscuring any visual information within the central triangle would disrupt the age estimation process. However, these studies did not specifically obscure the lower half of the face, and therefore the impact of wearing a facemask on age perception is not yet known.

Furthermore, individuals may also apply makeup, which, in addition to the facemask, could decrease age estimation accuracy further. The combined effect of makeup and masks on age perception is yet to be examined. As makeup to the eyeregion has been found to introduce more errors compared to other regions (Russell et al., 2019), makeup application to the eyes in combination with an absence of visible visual cues from the entire bottom half of the face, could result in further deterioration of age estimation accuracy. Our aim was to determine whether wearing a facemask or makeup affects age estimation accuracy, and whether pairing facemasks and makeup would further increase age estimation error. Specifically, we expected that makeup would increase the perception of age for young adult women, and that facemasks would impair age estimation; however, we did not predict whether the impairment would be biased towards an over or underestimation of age. Furthermore, we expected that the combined use of facemasks and makeup would yield a greater increase in overestimation bias compared to makeup alone, due to a reduction in facial age cues and enhancing of visible cues (eye region) through cosmetics.

## 2 METHOD

### 2.1 Participants

A total of 68 participants (57 female, 11 male) aged 18–65 years (mean = 36 years, SD = 13.81) volunteered in this experiment. Sixty‐five indicated that they were Caucasian, one Asian, one Hispanic and one selected ‘other’. Participants were recruited via social media and did not receive any monetary compensation for their participation. All reported normal or corrected‐to‐normal vision.

For the construction of the stimuli, 33 female participants volunteered. Of these, 27 were students from Bournemouth University aged 18–21 and six were recruited via social media aged 48–73. Participants received course credit or small monetary payment for their time. All volunteers were Caucasian except for three, two Chinese and one Mixed Caribbean. All the procedures were approved by the Ethics Committee at Bournemouth University.

### 2.2 Stimuli

Volunteers took passport‐style photographs of their faces in the four following ways: (1) no makeup and no mask, (2) makeup and a mask, (3) makeup and no mask and (4) no makeup and mask. Due to COVID‐19 government restrictions, participants could not attend the laboratory to have their photographs taken, therefore they were provided with detailed instructions to take the photographs within their own home. Photographs were taken using a digital camera or a phone camera and a digital timer or the assistance of a family member to steady the camera in front of the face. Participants were instructed not to take a ‘selfie’ with their arms extended to avoid distortion of facial features. Participants were instructed to stand in front of a white or cream background positioned facing towards a window with no shadows on the face, to remove headwear and glasses, to ensure hair was not covering the face, to take the photographs with a neutral facial expression, and to use a blue non‐medical mask for the masked photographs. For the makeup photographs, participants were instructed to apply makeup as though they *‘were attending an event’*, and to keep the same makeup for both makeup conditions (i.e. with and without a facemask). All participants wore foundation, and applied eyeshadow and mascara, and some, but not all, individuals applied eyeliner and eyebrow definition. Participants were provided with a checklist to self‐check the quality of their photographs. Participants also provided a unique pseudonym ID that they were asked to keep so that they may withdraw their image from the database and future use of the images if they decided.

**FIGURE 1:**
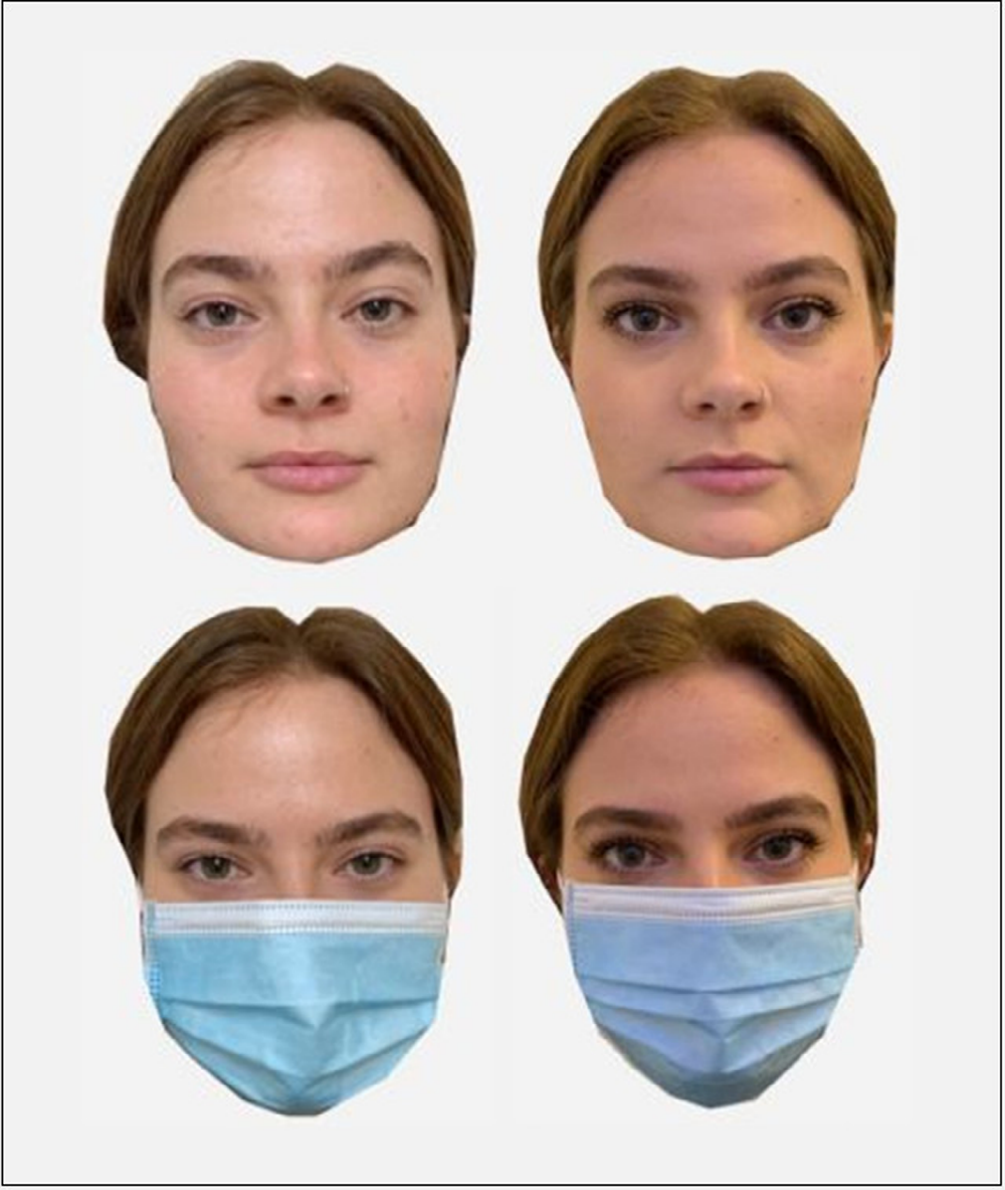
An example of one of the faces depicted with the four conditions of facemask (bottom) and makeup (right). The woman in the picture is 20 years old

The researcher screened all photographs, and five sets of images were excluded as they did not meet the criteria or were of insufficient quality. Therefore, a total of 28 photograph sets (112 images) were included in the experiment. All faces included in the final stimulus set were Caucasian except for three (two Chinese and one Mixed Caribbean). All images were cropped around the face to exclude the background and neck using GNU Image Manipulation Program (version 2.10) photo editing software (for an example, see Figure 1). All face images were resized to a width of 170 pixels and placed in the centre of the screen on a white background. The final stimulus set comprised 28 image sets, of these 22 sets included faces of women aged between 18 and 21, and six sets of women aged between 48 and 73. The focus of this study was to examine the effect of makeup and masks on young adults; however, the six sets of faces of older adult women were included to prevent observers from identifying a specific pattern (i.e. all young adults) and responding systematically during the experiment.

### 2.3 Procedure

Four versions of the experiment were created such that each observer only estimated the age of each given identity once, and all conditions of the identities (i.e. no makeup and no facemask/makeup and facemask/makeup and no facemask/no makeup and facemask) were counterbalanced across versions. Therefore, all observers estimated the ages of a total of 28 different identities comprising seven trials from each of the four conditions. The experiment was hosted on an online testing suite Testable (Rezlescu et al., 2020) with a restriction enabling observers to run the experiment on a laptop or computer, but not their phone or tablet. Observers were assigned randomly to one of the four versions. The experiment was calibrated to the size of the screen of the device using a standard approach in Testable. When the study commenced, observers were provided with instructions to estimate age as accurately as possible. Each trial began with a centre fixation cross for 800 ms, followed by the display of the face image for 2000 ms. When this time elapsed, the image was removed from view and replaced with a response box, observers were prompted to enter a two‐digit response using the number pad on the keyboard. There was no time pressure to enter their response, and observers pressed ‘enter’ when they were ready to proceed. All images were presented in random order, which was computer generated and different for each participant.

## 3 RESULTS

### 3.1 Data preparation

Estimation bias was measured by computing the estimation error by subtracting the estimated age from the actual age. These values were then averaged for each condition to create four average estimations for each observer. This was used to determine whether the faces were *overestimated* or *underestimated* for the four different conditions.^1^ Only the 22 trials comprising age estimates for the young adults aged 18–21 were included in these calculations and in the following analysis.

### 3.2 Individual differences in estimation bias

**FIGURE 2:**
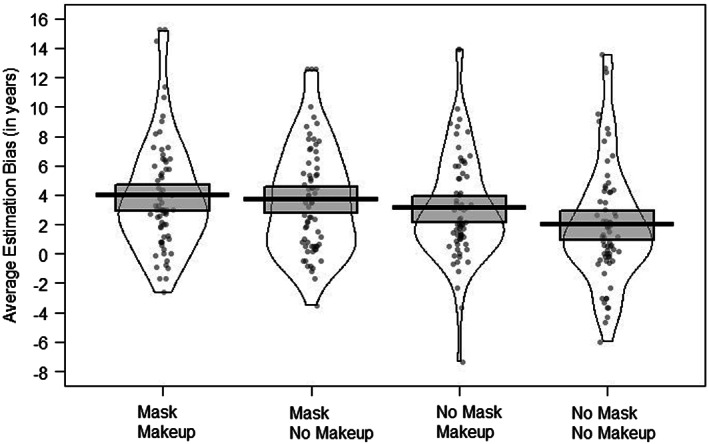
Observers' mean age estimation bias in years and distribution of raw scores when estimating the age of faces with or without a facemask, and with or without makeup. Positive scores reflect an overestimation of age and zero reflects no deviation from actual age. Band around the mean represents confidence intervals. The data here represents the non‐transformed means and data after outliers have been winsorized

First, we explored the range of age estimation bias on an individual level. When combining all conditions, this revealed a range of bias from an underestimation of 2.3 years and an overestimation of up to 15 years. These data show that only 12% of observers (8/68) were accurate within 1 year (±) of the actual age, while 78% (53/68) of observers overestimated by more than 1 year, and 10% (7/68) underestimated target age by more than a year. We also explored individual data for all four conditions separately. The distribution of scores is illustrated in Figure 2 and show a broad range of accuracy in age estimation. Of interest is the increase in number of observers overestimating age when women were presented with a facemask, makeup or both compared to the number of observers who underestimated the age of women without a facemask or makeup. Specifically, in the no mask/no makeup condition 16% (11/68) of observers underestimated the age of women by more than 1 year, compared to only 4% (3/68) in the mask/makeup condition and similarly in the mask/no makeup, and 6% (4/68) in the no mask/makeup. Furthermore, the largest average error made across all conditions was 20 years by two observers both in the mask and no‐makeup condition. Taken together, this indicates a greater tendency to overestimate the ages of faces wearing a facemask, makeup or both.

### 3.3 Estimation bias

Next, we compared age estimation bias for the four conditions. Data screening revealed four participants whose scores on one condition were identified as an outlier because their value was 3 SDs above or below the mean. Inspection of their data did not suggest that these extreme scores were not due to nonadherence of task instruction but that the participants were generally poorer at estimating age. While these data points are still valid, such extreme scores can have an undue influence on the analysis, therefore we decided to winsorize the values to the next highest estimate within the condition (Reifman & Keyton, 2010). These data are illustrated in Figure 2 and show that the ages of faces in all conditions were generally overestimated, however faces without any makeup or a facemask produced the least deviation from actual age (+2 years) compared to faces wearing a mask and makeup (+4 years). Furthermore, Normal QQ‐Plots suggested violation of normality in all conditions due to a slight positive skew most pronounced in the mask and makeup condition. Consequently, the data were transformed using an Aligned Rank Transformation (Kay & Wobbrock, 2019; Wobbrock, 2011) using ARTool package in R (version 4.0.3). The Aligned Rank Transformation provides an effective non‐parametric approach for multifactorial analysis, which, in contrast to other commonly used non‐parametric approaches, can effectively handle interactions (for a detailed comparison of approaches see Wobbrock, 2011).

To formally analyse these differences, a 2 (Face covering: mask, no mask) × 2 (Face Cosmetics: makeup, no makeup) non‐parametric repeated‐measures factorial ANOVA using the Aligned Rank Transformed data revealed a main effect of Face Covering, *F*(1, 201) = 22.06, *p* < .001, *ηp* ^2^ = 0.10, whereby the ages of faces without a facemask were estimated more closely to their actual age. The analysis also revealed a main effect of Face Cosmetics, *F*(1, 201) = 8.05, *p* = .005, *ηp* ^2^ = 0.04, such that the ages of faces without makeup were estimated more accurately. An interaction between the two factors was also found, *F*(1, 201) = 3.92, *p* = .049, *ηp* ^2^ = .02. To analyse this interaction, non‐parametric Wilcoxon signed‐rank *t*‐tests were performed with rank‐biserial correlation coefficients reported as effect sizes. These results are summarised in Table 1 and suggest that both facemasks and makeup have an impact on estimation bias with small to medium effect sizes, however a combination of the two is not additive.

**TABLE 1:** Summary of Wilcoxon signed‐rank post hoc comparisons with rank‐biserial correlation coefficients by participant reported as effect sizes

		*W*	*p*	Rank‐Biserial correlation
Mask	Makeup versus no makeup	1276.5	.184	0.19
**No mask**	**Makeup versus no makeup**	**1622**	**.006**	**0.38**
Makeup	Mask versus no mask	1549	.022	0.32
**No makeup**	**Mask versus no mask**	**1750**	**<.001**	**0.49**

*Note*: Bonferroni adjustment was used for multiple comparisons (*α* is 0.05/4 = 0.0125). Comparisons in bold denote significance (*p* < .0125).

### 3.4 Correlations for estimation bias

We also explored whether the age of the observer was correlated with the estimated age of the face for the four different conditions. Due to non‐normal distribution, a non‐parametric spearman's correlation was performed for all four conditions. This analysis revealed a positive correlation for all conditions, all *r*_s_ ≥ 0.34, all *p*s ≤ 0.005, with the exception of faces with no mask and no makeup, *r*_s_(67) = 0.21, *p* = .09. In summary, older observers overestimated faces in the three manipulation conditions to a greater degree than younger observers, but this pattern did not persist for unmasked faces with no makeup.

## 4 DISCUSSION

This is the first study to investigate the effect of facemasks, and the combined effect of wearing a facemask with makeup, on the perception of age of young adult female faces. Faces across all four conditions were perceived as older than their actual age. Overestimation was greater for faces wearing a facemask or wearing makeup, but contrary to our prediction, a combination of both did not impair performance further. The findings also revealed a large range of individual differences in age estimation ability across all conditions.

In line with previous findings, 18‐ to 21‐year‐olds were perceived to be older than their actual age by at least 1 year (Thorley, 2021; Willner & Rowe, 2001) and this upward bias increased with the application of makeup (Russell et al., 2019). The reason for this upward bias effect of makeup on young faces is not fully understood, and contrasts with the more youthful appearance of older people wearing makeup (Russell et al., 2019). Although older faces appear more youthful because of feature size manipulation (e.g. making eyes appear larger), increasing feature contrast, and enhancing skin homogeneity (Russell et al., 2019), enhancing these features in younger women does not reduce their perceived age. Rather, it has been suggested that makeup in younger women acts as a visual contextual cue activating judgements influenced by beliefs about social norms, specifically that makeup use is associated with adulthood (Russell et al., 2019).

In the present study, we also examined the effect of facemasks on age estimation accuracy. Masked faces were estimated to be approximately 4 years older than their actual age compared to 2 years older when unmasked. We note that the average error of 4 years falls within the range of error found in some other studies (e.g. Clifford et al., 2018; Dehon & Brédart, 2001; Sörqvist & Eriksson, 2007), and by comparison the effect of facemasks on age estimation may not appear large. However, these studies differ methodologically (for example, use of time pressure versus self‐paced, accessories on face and hair, average age of perceivers) and consequently the tasks also differ in difficulty. Therefore, such direct comparisons of error across studies offer limited insight.

Although previous research suggests that the eye region is an important facial feature for accurate age judgements (Jones & Smith, 1984), disrupting the processing of other facial features while keeping the eye region intact also impaired age estimation accuracy in this study. However, this raises the question of whether the pattern found here is driven by an individual facial feature obscured by the mask, a combination of features, or the disruption of the central triangle of the face. Few studies have examined the role of individual features in age perception (Jones & Smith, 1984; Liao et al., 2020). Of these, it was found that children aged 3–9 made more errors during the ranking of adult faces by age when the eye region was masked, compared to when the faces were unmasked, or when a combination of features were masked (mouth and chin, or face shape) (Jones & Smith, 1984). The masked nose and cheek condition also produced more errors than the other conditions, though not to the same extent as the masked eye region. In a recent eye‐tracking study, observers directed their gaze towards the central triangle of the face both when viewing adult faces freely and when tasked to estimate ages, however in the tasked condition, eye gaze was also redirected towards the lower part of the face (Liao et al., 2020). Both these studies suggest that the bottom half of the face is important for age estimation, and our findings add further support. The present study is a first step for demonstrating the impact of facemasks on age perception; however, the theoretical mechanism underlying this effect is beyond the scope of this paper and a systematic examination of different facial features on age perception is an important avenue for further investigation.

We also examined the combined effect of makeup and facemasks. Results indicate that wearing makeup or a facemask individually increased the upward bias by an average of 1.2 and 1.7 years, respectively. However, estimation bias did not increase further when facemasks and makeup were combined. This finding is contrary to our prediction that wearing a facemask *and* applying makeup would increase error bias even further given the pertinent role of makeup to the eyeregion compared to other facial features (Russell et al., 2019). To increase ecological validity, the style and intensity of makeup was left to participants to apply in a manner that was natural for them to wear to a formal event. For this reason, we did not control for the intensity of makeup applied to faces. Although research has not found a difference in age estimation bias when using different makeup intensities (Russell et al., 2019), it is plausible that makeup intensity to the eye region, when paired with a facemask, could influence age estimation differently due to a reduction on reliance from other visual cues for age judgements.

This study also explored age estimation ability at an individual level. Findings demonstrate that there is a broad range in ability, while some individuals were fairly accurate in their estimates (within = ±1 year), others provided estimates with a deviation of up to 20 years over the target's actual age. Furthermore, older observers overestimated faces to a greater degree when the faces were wearing a mask, makeup, or both, but this pattern did not reach significance when the face was without makeup or mask. These findings support previous research suggesting that the perceiver's age influences age estimation from faces in two ways. First, studies have reported a general decline in age estimation accuracy linked to cognitive ability for older participants compared to younger participants (Voelkle et al., 2012). Secondly, an own‐age bias has also been previously recorded, demonstrating an advantage for estimating ages of faces similar to that of the observer (Moyse et al. (2014); Willner & Rowe, 2001) but this pattern is not consistent across studies (Burt & Perrett, 1995). Further exploratory analysis for perceiver age bias in the present study is provided in the supplementary materials, this analysis indicates that perceivers over the age of 34 produced greater error in their estimates of 4.4 years compared to 1.8 years by perceivers under the age of 34 across all conditions. As this study did not set out to investigate own‐age bias and therefore did not include the older target faces for a comparison group, it is not possible to conclude whether perceiver age bias found here is due to age related decline or an effect of own‐age bias.

Other group characteristics may also account for some of the variance recorded in the present study, namely gender and ethnicity of stimulus and participants. Although these characteristics have not been extensively documented, existing findings suggest that Caucasian participants perform better when evaluating Caucasian faces (Dehon & Brédart, 2001; Thorley, 2021), but this own‐ethnicity bias does not persist for African participants (Dehon & Brédart, 2001). Few studies have examined own‐gender bias in age estimation from faces, and those which have did not find evidence to support this (Dehon & Brédart, 2001; Voelkle et al., 2012). The present study did not examine gender and ethnicity bias, however this may warrant further investigation.

Despite these findings, there are some limitations to consider. Only female faces were used to examine the interaction of makeup and facemasks. Some studies report an advantage for accuracy of male faces compared to female faces (Dehon & Brédart, 2001; Voelkle et al., 2012) which may be due to women being more likely to apply cosmetics and makeup to their faces (87% of women compared to 7% of men reported wearing makeup at least once in a government survey; Waldersee, 2019). It is therefore not known whether facemasks and makeup affect the perceived age of men and women differently. Additionally, to control for extraneous variables we did not include the rest of the body in this experiment. While faces have been found to provide the strongest cue for age leading to least errors (Cattaneo et al., 2009), additional visual cues from the body, such as body height and shape, could be used by the assessor to provide a more accurate estimate.

Finally, this study focussed on young women aged 18–21, although it is not inconceivable to expect similar patterns with 17‐year‐old women considering the ages of 13‐ and 16‐year‐old girls also tend to be overestimated under ‘normal’ conditions (Willner & Rowe, 2001). However, face age estimation research with adolescence remains scarce and further work examining the effect of facemasks and makeup with adolescent faces is warranted. Nonetheless, the findings with the 18‐ to 21‐year age group are relevant still. Although the most common minimum purchasing age in the European region is 18 years old, minimum age restrictions vary widely across countries and in some cases also depend on the item of sale (for example, beverage type for alcohol sales). Specifically, for example, in Norway and Sweden, the age limit for beer and wine is 18, but rises to 20 for sales of spirits (Kadiri, 2014), and in some states of the United States of America, Sri Lanka, and Egypt, the law restricts sale of alcohol to individuals aged 21 and over (International Alliance for Responsible Drinking [IARD], 2020).

In conclusion, these findings offer some practical implications, particularly in the context of age estimation in the sales of restricted items. Although the *‘Challenge 25’* policy is in place in the United Kingdom to encourage sales personnel to verify identification for individuals appearing under the age of 25, many countries do not implement a similar retail sales strategy. Even with such a policy in place, the wide range in estimation ability with some individuals (specifically, 12% on average for neutral faces and between 13% and 20% on average for faces wearing a makeup, a facemask, or both) who overestimated age by 7 or more years, suggests that a percentage of young women may still be incorrectly perceived to be older than 25. The *‘Challenge 25’* policy therefore goes some way to mitigate risk of sales to minors but does not eliminate the potential for decisional errors among those whose age estimation skills are poor, and the potential for error increases further when faces are partially obscured or transformed with the application of makeup.

Finally, the range of individual ability found here also indicates broader implications extending to settings which require security personnel to make age judgements. For example, for the classification of children and adults of undocumented refugees by immigration officers or for the identification of minors in the assessment of online illicit content by internet safety officers. In such situations, it may be beneficial to delegate such tasks to groups of people who are proficient in the task, though, further work to understand individual differences is needed and we hope that these findings will prompt further research in this field.

## CONFLICT OF INTEREST

The authors declare no conflict of interest.

## Supporting information


**Appendix** S1: Supporting information

## Data Availability

The data of this study are available upon request from the corresponding author.

## Endnote

^1^
Absolute error, which reflects average deviation from actual age without direction, was also calculated and analysed separately for completion (See Supplementary Materials).
