# Supplementary material for: Your ID, please? The effect of facemasks and makeup on perceptions of age of young adult female faces
Source: Appl Cogn Psychol. 2022 Feb 6;36(2):453–9. doi: 10.1002/acp.3923 (PMC9011877; doi:10.1002/acp.3923)
Supplement: Supplementary file 1 — Appendix S1: Supporting information [file ACP-36-453-s001.docx]

Your ID, please? The effect of Facemasks and Makeup on Perceptions of Age of Young Adult Female Faces

**Online Supplementary Materials**

**Absolute Error**

*Absolute Accuracy,* which reflects the overall extent of mis-estimation by averaging deviation from actual age and discounting direction, was also calculated (see Vestlund et al., 2009). For the calculation of the absolute error, all the values for the age estimation error for each trial were transformed to a positive value. The average for each condition and observer was then computed from these values. Data screening revealed nine outliers, as in the previous analysis their value was trimmed to the next highest value within the condition. Normal QQ-Plots suggested strong violation of normality in all conditions, and therefore the data were transformed using an Aligned Rank Transformation. A 2 (Face covering: mask, no mask) x 2 (Face Cosmetics: makeup, no makeup) non-parametric repeated-measures ANOVA revealed a main effect of Face Covering, *F*(1, 201) = 5.54, *p* < 0.01, ŋp^2^ = 0.03, whereby faces with a mask produced greater error compared to faces without a mask. No main effect of makeup, *F*(1, 201) = 0.07, *p* = 0.80, ŋp^2^ = 0.0003, or interaction, *F*(1, 201) = 0.38, *p* = 0.54, ŋp^2^ = 0.002, were found.

**Age differences in Estimation Bias**

To explore whether there were differences in accuracy depending on the age of the perceiver a median split was performed to evenly divide the sample into two groups. Therefore, the sample was grouped by those aged under 34 (N = 32), and those age over 34 years (N = 36). A 2 (Perceiver age group: under 34, over 34 years) x 4 (Condition: Mask/makeup, make/no makeup, no mask/makeup, no mask/no makeup) mixed factorial ANOVA was performed. This revealed a main effect of mask, *F*(1, 66) = 20.24, *p* < 0.001, ƞp^2^ = 0.24, whereby faces with masks were estimated to be 3.8 years older than their actual age compared to 2.6 years for faces without masks. A main effect of makeup, *F*(1, 66) = 8.22, *p* = 0.006, ƞp^2^ = 0.11, whereby faces with makeup were perceived to be 3.6 years older compared to 2.8 years for faces without makeup . A main effect of perceiver age was also found, *F*(1, 66) = 12.67, *p* < 0.001, ƞp^2^ = 0.16, such that perceivers over the age of 34 produced greater overestimations of 4.4 years compared to those under the age of 34 who overestimated the faces on average by 1.8 years. No interactions were found, all *Fs* ≤ 0.69, all *p*s ≥ 0.41.

**REFERENCES**

Vestlund, J., Langeborg, L., Sörqvist, P., & Eriksson, M. (2009). Experts on age estimation. *Scandinavian Journal of Psychology*, *50*, 301–307. <https://doi.org/10.1111/j.1467-9450.2009.00726.x>

|  |
| --- |
